# Proceedings of the American Medical Association

**Published:** 1850-11

**Authors:** 


### PROCEEDINGS OF THE AMERICAN MEDICAL ASSOCIATION.

We have received the proceedings of the American Medical Association, which met in Cincinnati in May last. These proceedings make a respectable sized volume, but the generality of the Reports made on the occasion are less interesting than those of preceding years. The proceedings of 1849 were highly instructive, and honorable to American medicine. But the reports in the present volume are far from being as full and instructive as they should have been. The materials for filling these reports were ample, and should have been used for the advancement of the profession. The report upon medical literature must be excepted, however, from these censures; it is an excellent and valuable contribution towards the elevation of medical science.

The annual convocation of the medical men of the Union is remarkably well adapted to the improvement of the profession generally, and we hope to see its influence extend until the entire profession feels the force of the genial current, and is carried upward to that position it should occupy in public estimation. We are confident that ample remedies for the evils under which the medical profession labors are in the hands of the members of the profession, and it is never too soon to commence the removal of abuses, and the destruction of evils. It is in this, as in the matters described by Junius: "that which is custom becomes a rule, and the precedent of to-day is law to-morrow." The elevation of medical excellence, the thorough preparation of men for the arduous and responsible duties of practitioners of medicine, the suppression of quackery, and everything that can tend to honor and elevate the laborers in medical science, are at the command of the profession, and should be used for the attainment of what all good men hope for, but in the fruition of which, few have faith. B.

